# Electricity and Dentistry

**Published:** 1897-10

**Authors:** James L. Gethins

**Affiliations:** Boston, Mass.


### ELECTRICITY AND DENTISTRY.[1]

BY JAMES L. GETHINS, BOSTON, MASS.

In addressing the Massachusetts Dental Society on this subject I feel like saying "Brother electricians," as I know you are well versed in the art. You all know how useful electricity is in dentistry. You see it in the dental engine, mallet, mouth-lamp, and in the much-discussed subject of cataphoresis. The question of using electric batteries in dental work has been considered and discussed, and seems to have been quite successful in some instances, while in others it has been a failure. In fact, I think I may say in a general way that electric batteries have received quite a black eye in the profession. With an experience of twelve or fifteen years the most successful thing is the storage battery, because you have very low internal resistance, and you have practically a reservoir of electricity, and the voltage is utilized in the external circuit instead of being wasted in the battery itself.

The question of the batteries, their value in proportion to the cost of maintenance, and the amount of care they require is important. We can use bichromates and acids, but these batteries are condemned because difficult to handle. There is one question that has been often put in reference to this, and I am surprised to find in the text-books that the objection to the use of batteries has been the cost of zinc. That is all wrong. Zinc is very inexpensive, comparatively speaking. From two pounds of zinc we can get practically two horse-power at a cost of about ten cents. The practical difference in cost between using a dynamo to produce

---

[1] Read before the Massachusetts Dental Society, Boston, June 2, 1897.

current and electric batteries is in proportion of about twenty-five to one. For this light class of work, running dental engines, mouth-lamps, cataphoresis, etc., storage batteries are successfully used.

With reference to the subject of cataphoresis, I have recently made several tests, and was somewhat surprised with the result. Different concerns are putting out apparatus involving all the way from five cells of battery to fifty or sixty. I recently made a test in a doctor's office in this city, and the doctor's apparatus was successfully used for this very purpose,—cataphoresis. He had what he called the volt-selector. It was an apparatus that struck me as unique, for the volt-selector moved rather mechanically. I did not think that indicated the actual pressure. I said, "Suppose we put the voltmeter on and see what we get." The result of that test was that I found when I moved the handle round, whether we had the battery current on or not, it indicated so many volts pressure. I wish to advocate very strongly in all transactions of that kind an accurate-reading milliameter, and I think this question of cataphoresis resolves itself into this. It certainly is not the voltage that accomplishes the work, as sufficient voltage to overcome the resistance only is necessary. It is the actual quantity of current. It is a case of osmosis, pure and simple, and the idea of building up a high voltage, and getting one volt at the tooth when there are forty-four at the outside circuit, strikes me as absurd.

I suppose, of course, that you are familiar with Ohm's law, but I find that in practice it is the gentlemen who are not using it every day who become confused. You must always remember that the volts indicate pressure, and the volts and ampères are regulated by resistance.

Now, I met another friend in the profession the other day, and he had what might be called a volt-selector, with simply ten cells of battery, and a switch in connection with it, so that he could use one cell at a time. That gave him one volt under the full strength of the battery. He found that five or six were all that were necessary to accomplish the desired result in from ten to fifteen minutes. The objection to that battery was that in removing the switch from one point to another the connection was broken, and the patient received a shock. His apparatus was very crude, but it appeared to me that he was getting down to first principles; he was actually finding how many volts he wanted at the tooth, and how much battery was required to do this work.

Now, I will give you a little illustration as to what this machine will do. We have on this dental engine four volts, and for that matter we could construct a dental engine so that it would run with two. We do not require fifty or one hundred or one hundred and ten. I brought this to show that the current and not the voltage does the work. [Mr. Gethins here gave an illustration of the running of the dental engine.]

When I use the cautery-knife it takes about twenty ampères of the current. [Mr. Gethins here illustrated the use of the cautery-knife.] That illustrates the point I wish to get at. That has really taken about twenty ampères of current to develop that intense heat which you notice. There are only four volts back of it, and it could be done with two, and all of this kind of work can be done with a very small storage battery. Probably the actual voltage I am using there now is about three. The principle of cataphoresis is somewhat similar in effect to the formation of a storage battery. We take the lead plates and apply the current to them in the battery, we oxidize one plate and deoxidize the other by the passage of the electric current. It does not make a particle of difference whether I have ten thousand volts back of that amount of current or only two and a half volts, I get the same effect on these lead plates. It is the electrical action which takes place, and, of course, it is a question of the actual quantity of the current.

I think, in a general way, on this subject of cataphoresis that it would be very well, indeed, if the practical electrician and the dentist should get together and experiment to find out exactly how much current is required. In fact, in looking this matter up, I have ascertained in a great many cases that it has taken about four-tenths of one milliampère,—that is, a pressure of one volt with twenty-five hundred ohms resistance. That is a minute current which we are using in this service, and, as I have stated before, I think if I were advising the use of any apparatus, I should test it very carefully, and be sure to know what I was doing, because I know that the dentists do not know what the effect would be of too much current.

